# 1181. Evaluation of the Safety of a Beta-Lactam Allergy Protocol

**DOI:** 10.1093/ofid/ofad500.1021

**Published:** 2023-11-27

**Authors:** Alexandra K Sakowski, Jaime Borkowski, Karen Rhodes

**Affiliations:** Northwestern Medicine Delnor Hospital, St. Charles, Illinois; NM Delnor Hospital, Geneva, Illinois; Delnor Hospital, Geneva, Illinois

## Abstract

**Background:**

Beta-lactam allergies (BLA) pose a challenge to the optimization of antibiotic therapy as some of these are not true allergies, are minor reactions, or instead may be adverse effects. This issue is further complicated by the fact that traditional methods of challenging allergies can be costly or time-consuming. The purpose of this study was to evaluate the safety of a protocol which directly challenges patients with a BLA via full dose administration of the challenge antibiotic based on the risk of cross-reactivity.

**Methods:**

This retrospective, observational, case-control study compared the incidence of hypersensitivity reactions following the administration of a beta-lactam antibiotic in patients with a reported BLA to that of those without a reported BLA. It included adults in the community hospital setting who received a beta-lactam antibiotic for any indication during an inpatient admission between March 2018 and October 2022. The treatment groups were separated by the presence or absence of a documented BLA. The primary outcome was the occurrence of a hypersensitivity reaction following the administration of a beta-lactam antibiotic.

**Results:**

A total of 910 out of 1000 patients were eligible for evaluation, and 116 of those patients had a history of a BLA documented on the chart whereas 794 did not. The primary outcome of an IgE-mediated hypersensitivity reaction occurred in 1 out of 116 in the BLA group and 4 out of 794 in the non-BLA group (OR 1.72; 95% CI 0.19-15.5) (see table).

Results

**Figure:**
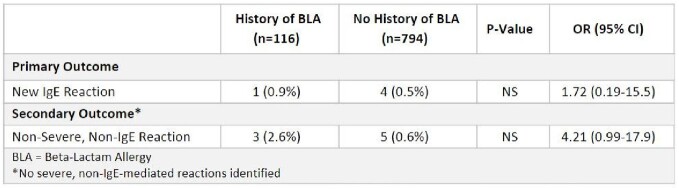

**Conclusion:**

No significant increase in the incidence of IgE-mediated allergic reactions was noted in the patients with a history of a BLA, however, the study was not powered to detect a difference due to the rarity of IgE-mediated reactions. Despite a trend towards an increased risk of reactions in patients with a history of a BLA, the majority of the few reactions that occurred were non-severe in nature. The benefits of challenging a BLA outweigh the risks in this setting.

**Disclosures:**

**All Authors**: No reported disclosures

